# Exploring Learner Engagement With Multiple Sources of Feedback on L2 Writing Across Genres

**DOI:** 10.3389/fpsyg.2021.758867

**Published:** 2021-10-15

**Authors:** Yali Shi

**Affiliations:** Department of English, School of Foreign Studies, Jiangnan University, Wuxi, China

**Keywords:** learner engagement, feedback, multiple sources, L2 writing, genres

## Abstract

While there have been growing amount of research on learner engagement with feedback on Second Language (L2) writing in the past decade, learners' multi-dimensional engagement with feedback from multiple sources across different genres has remained under-explored. To address the gaps, this study investigated how six second-year English majors engaged behaviorally, cognitively and affectively with automated, peer and teacher feedback across three genres (argumentation, exposition, and narration) in an L2 writing class given online over a 16-week semester. Through the textual analysis of learners' drafts, feedback and revision and qualitative analysis of their interview transcripts, it was found that the quantity and incorporation rate of feedback in general, across three feedback sources and two feedback types all differed by genres; surface-level teacher feedback remained the most highly incorporated, though. Learners' engagement with feedback also varied, suggesting its complexity triggered by the mediating effect of contextual and individual factors plus the interconnectedness and inconsistencies among engagement dimensions. Two pedagogical implications were provided to enhance learner engagement.

## Introduction

As a key concern in L2 writing, feedback has been widely studied in L2 writing research. While earlier studies focused on the effect of feedback on quality of writing products (e.g., Nelson and Schunn, 2009), learner engagement with feedback has received growing attention during the past decade or so. It is conceptualized as a multi-dimensional construct which is shaped by both contextual and learner factors (Fredricks et al., 2004; Ellis, 2010). Drawing on the research on L2 writing, SLA, and education, studies on learner engagement with feedback have provided empirical evidence for its “multifaceted, contextualized, temporal and individual-based nature” (Han and Gao, 2021, p. 56). The findings shed light on how learners process and use the feedback during revision and thus provide insights into the way to enhance their engagement and learning.

However, previous literature displays three features, which suggest research gaps to be filled. First, most studies have taken one or two perspectives (e.g., Bai and Hu, 2017; Sánchez-Naranjo, 2019; Zhang, 2020), with only several approaching to it multi-dimensionally (e.g., Han and Hyland, 2015; Zhang, 2016; Zhang and Hyland, 2018; Zheng and Yu, 2018; Fan and Xu, 2020; Koltovskaia, 2020). In addition, with the availability of multiple sources of feedback for learners nowadays, there are several studies on learner engagement with various feedback combinations (i.e., automated and teacher feedback, automated and peer feedback, and teacher and peer feedback) (e.g., Lai, 2010; Dikli and Bleyle, 2014; Dressler et al., 2019), yet those on learner engagement with feedback from multiple sources have been fewer (e.g., Tian and Zhou, 2020). Moreover, although the role of task genre in the effectiveness of feedback has been brought to attention (Stevenson and Phakiti, 2014) and also identified (Lv et al., 2021), little attention has been paid to the effect of genre on learner engagement with feedback.

To address these gaps and to gain further insights into the nature of learner engagement with multiple feedback sources across genres, this study, established on Han and Hyland (2015)'s multi-dimensional framework of learner engagement, attempts to explore how six second-year English majors engaged behaviorally, cognitively and affectively with automated, peer and teacher feedback across three genres (i.e., argumentation, exposition, and narration) in a 16-week L2 writing course. Specifically, there are two research questions.

1) To what extent did automated feedback, peer feedback and teacher feedback differ in frequency and uptake rate across genres?
2) How did learners engage behaviorally, cognitively and affectively with the three feedback sources across genres?

## Literature Review

### The Concept of Learner Engagement With Feedback

Originally as a concept in education, learner engagement refers to learners' commitment to learning. Fredricks et al. (2004) proposed a multifaceted concept of engagement, which consists of behavioral engagement, cognitive engagement, and emotional engagement. Ellis (2010) introduced this concept to the study of oral and written corrective feedback (CF) in second language acquisition and defined engagement as how learners respond to the feedback, which can be viewed from whether and how learners incorporate CF, how they pay attention to CF, and how they respond affectively to CF. As emphasized in both frameworks, learner engagement doesn't take place in vacuum but under the influence of both contextual (e.g., task characteristics, teaching settings) and individual (e.g., learning belief, personality) factors. Han and Hyland (2015) made some finer-grained adaptations of Ellis (2010)'s and proposed a multi-dimensional framework of learner engagement with written CF (WCF) in particular. Specifically, behavioral engagement includes revision operations and revision strategies; noticing, understanding, cognitive operations and meta-cognitive strategies constitute cognitive engagement; affective engagement encompasses emotional changes during the revision process as well as attitudes and immediate emotional reactions. Taken together, to explore learner engagement, it is necessary to not only take multiple perspectives but also attend to contextual and individual factors which condition its intensity and manner. The purpose is to make clear how engagement works in order to shed light on how to enhance it and thereby to facilitate learning.

### Research on Learner Engagement With Feedback in L2 Writing

During the past decade or so, there have been a number of studies on learner engagement with single-source (i.e., teacher, peer or automated feedback) or multiple-source feedback in L2 writing. Yet most of them dealt with one or two aspects of engagement.

Learner engagement with teacher feedback was approached in some studies from a single perspective, such as learners' cognitive processing (Crosthwaite et al., 2020), emotional responses (Mahfoodh, 2017; Han and Hyland, 2019), or quantity and quality of revision (Ferris, 1997; Hyland, 2003). Others explored contextual (e.g., feedback features, tasks) and individual factors (e.g., language ability, learner beliefs) which might influence learner engagement (Han, 2017, 2019). Only two took the multi-dimensional approach. Specifically, Han and Hyland (2015) found that when engaging with teacher WCF, four L2 college learners' revisions, cognitive and meta-cognitive operations and emotional responses were influenced by their L2 learning beliefs, experiences, goals, and writing ability. The other study (Zheng and Yu, 2018) revealed that for lower-proficiency English majors, the adequacy of their understanding of teacher WCF, cognitive operations and use of meta-cognitive strategies was limited by their lower language proficiency, despite their positive emotional engagement.

While some studies probed into the quantity and quality of peer feedback by peer reviewers of different language proficiency levels (Wu, 2019), those on learners' engagement with peer feedback are relatively few. Except some exploring learners' cognitive processing (e.g., Sánchez-Naranjo, 2019), two studies examined it in a multi-dimensional manner. One (Fan and Xu, 2020), which was situated in the college English class, found that for the intermediate-level college EFL learners, form-focused peer feedback elicited more positive emotional engagement and more extensive and deeper behavioral and cognitive engagement than content-focused one. In contrast, different results came from the other study (Saeli and Cheng, 2021), which was in the context of the TOEFL-iBT-preparation class for upper-intermediate and advanced undergraduate EFL learners. That is, it was content-related feedback rather than grammar-related one that elicited positive affective engagement, which in turn led to active engagement behaviorally and cognitively. As to learner engagement with automated feedback, some explored it mainly in terms of learners' revision (e.g., frequency, types, quality) which was influenced by both individual (e.g., language proficiency, learner beliefs) and contextual factors (e.g., feedback focus, learning contexts) (Li et al., 2015; Bai and Hu, 2017; Zhang, 2020). Two others provided a multidimensional examination and revealed tricky interconnections between dimensions of engagement for L2 undergraduate learners. Specifically, Zhang (2016) found that behavioral engagement was both prompted by cognitive processing and influenced by emotional responses. Yet the other study (Koltovskaia, 2020) revealed that behavioral engagement could be realized without adequate cognitive processing.

In addition, there are studies comparing learner engagement with different sources of feedback. For example, among those on automated feedback used together with teacher feedback (Dikli and Bleyle, 2014; Wilson and Czik, 2016), it was found that despite its less accuracy, learners overall thought positively of automated feedback when it was combined with teacher feedback. Another study (Zhang and Hyland, 2018) revealed that learner engagement with these two feedback sources differed in revision operations, attitudes, and the use of cognitive and meta-cognitive strategies. When automated feedback and peer feedback were used together, it was found that undergraduate EFL learners incorporated peer feedback significantly more frequently, who highly valued peer feedback and considered automated feedback somewhat vague (Lai, 2010). As to the combination of peer and teacher feedback, the latter was generally more frequently incorporated and more highly valued (Yang et al., 2006; Zhao, 2010; Dressler et al., 2019). So far only one study (Tian and Zhou, 2020) involved three feedback sources, which examined how five Chinese EFL learners engaged with them on three essay genres in an on-line college English writing course. Results uncovered learners' dynamic and reciprocal engagement across tasks, which was mediated by contextual and individual factors.

Despite these illuminating findings on learner engagement with feedback, there are three gaps in the existing studies. To begin with, studies exploring it from multiple dimensions were relatively fewer (e.g., Han and Hyland, 2015; Zhang, 2016; Zhang and Hyland, 2018; Zheng and Yu, 2018; Fan and Xu, 2020; Koltovskaia, 2020). Besides, those on how learners engage with feedback from multiple sources are far from being enough (Tian and Zhou, 2020). More importantly, genre, as a key task characteristic, has been less attended to and its effect on engagement has remained under-explored. There is no denying that some studies included more than one genre (e.g., Hyland, 2003; Lai, 2010; Zhao, 2010; Zhang, 2016; Mahfoodh, 2017), but different genres were treated as a whole without making distinctions. Even if some comparisons in learner engagement across genres were provided (e.g., Li et al., 2015; Sánchez-Naranjo, 2019; Tian and Zhou, 2020), the genre was addressed more as a longitudinal factor to indicate the change of learner engagement over time. As Stevenson and Phakiti (2014) added the systematic examination of genre influence to the to-do list of the research on the effectiveness of automated feedback, the same is reasonably true for that on learner engagement with multiple sources of feedback. Also, since it was found by a meta-analysis (Lv et al., 2021) that task genre plays a mitigating role in the effect of online feedback on L2 writing quality, one may wonder whether it also plays a role in learner engagement with feedback. Therefore, to fill the gaps, this study aims to investigate learners' behavioral, cognitive and affective engagement with automated, peer and teacher feedback across argumentation, exposition, and narration.

## Methods

### Research Context

The study was situated in two English Writing II classes for second-year English majors in a Chinese university. It is devoted to essay writing of different genres (i.e., argumentative, expository, and narrative), while English Writing I focuses on words, sentences and paragraph writing. The two classes (24 and 26 students) each met online^1^ once a week for 90 min over the 16-week semester in the spring of 2020, taught by the same teacher.

The instruction for this course is both genre-based and process-oriented. Students' drafts undergo three feedback-revision cycles. Specifically, in the first cycle, students revise their first drafts based on automated feedback. The second cycle follows in which they make revisions of their second drafts based on feedback given by their peers. The process ends with the submission of the final drafts after revisions made on the basis of teacher feedback in the third cycle.

In this study, automated feedback was provided by *Pigai*^2^, an Automated Writing Evaluation system widely used in Chinese universities since its launch in 2011. It provides holistic scores, overall comments, as well as sentence-based CF on grammar, vocabulary, mechanics, and collocation immediately after learners submit their drafts. Moreover, on *Pigai*, the teacher assigned students randomly to offer feedback on each other's drafts and then offered feedback on students' revised drafts. The system keeps a record of every piece of feedback and its corresponding revision in each version of the drafts.

### Participants

Maximum variation sampling (Dörnyei, 2007) was applied in that a higher-proficiency student, an average one, and a lower-proficiency one were recruited from each of the two classes, totaling six (Table 1). They were selected based on (1) their English language test performance in previous semesters, (2) their teacher's recommendations, and (3) their willingness to participate.

**Table 1:** Information about the participants.

**Language proficiency**	Higher		Average		Lower	
**Participants**	Liu	Wang	Yang	Yao	Ge	Ren
**Gender**	F	F	F	M	F	F

### Data Collection

**Figure 1:**
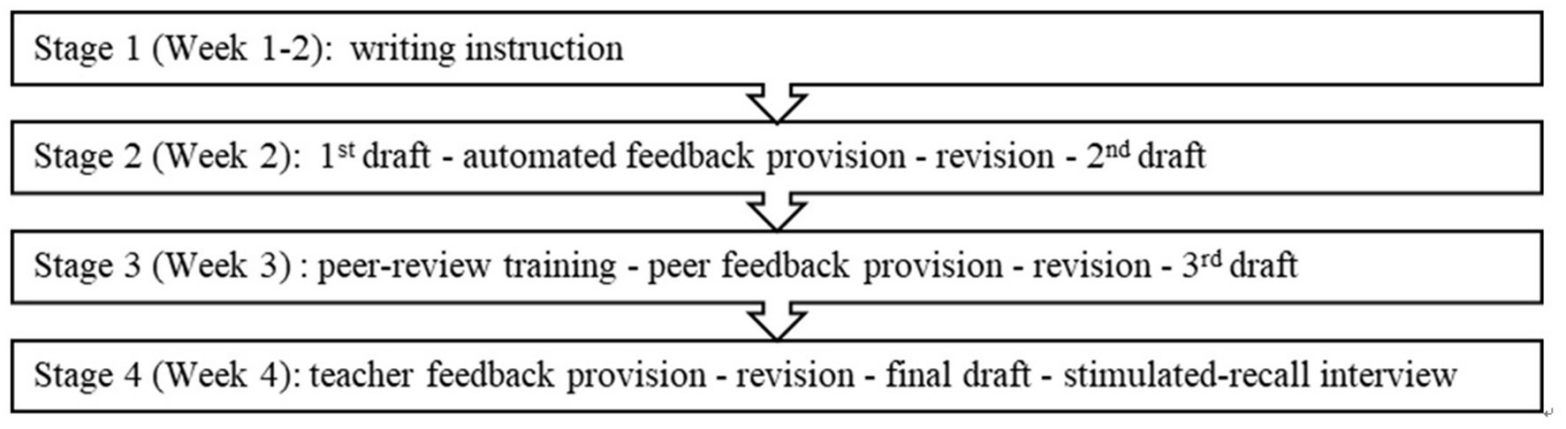
Data collection procedures for each genre.

Data collection started from Week 3 of the semester which began with the course orientation in Week 1, followed by the introduction of essay writing in general in Week 2. In course orientation, besides informing students about the course content and the multi-draft writing process based on feedback from multiple sources, the teacher also introduced *Pigai* to the students and how to use it. From Week 3 on, writing of three genres was addressed in turn. For each of the genres, four-week data collection involved four major stages based on three feedback-revision cycles, as illustrated in Figure 1.

At the end of each cycle, the participants were each given a stimulated-recall interview (see Appendix 1 for the interview outline) at a time of their own choices. The permission of audio-recording was obtained after they were informed about the research purpose. The interview was conducted in their native language and audio-recorded by the researcher. During the interview, participants' drafts with the feedback points and revisions were used as the stimulus to support their recall of what was going on in their minds when they were making revisions (or not). The duration of interviews ranged from 15 to 25 min.

Finally, at the end of the semester, the participants were each interviewed on their general evaluations of three feedback sources across genres. Two types of data were thus collected for this study: a total of 146 drafts with feedback and revisions, and audio-recordings of 24 interviews (about 390 min in total).

### Data Analysis

Data analysis consisted of text analysis of participants' drafts and qualitative analysis of interview transcripts.

To answer Research Question 1, essay drafts were given a detailed analysis in terms of the feedback quantity, type, and uptake rate. Feedback quantity was determined based on the identification of each feedback point. Evaluative feedback and information-oriented feedback (e.g., synonym suggestion, word frequency) were not considered for they do not require revision. Feedback type was coded based on Tian and Zhou (2020)'s coding scheme which classified feedback into surface-level and meaning-level categories at different levels (Table 2)^3^. Finally, if a given feedback point led to the corresponding revision at each feedback-revision cycle, it was categorized as incorporated. If no revision was found, the feedback was not incorporated. Then the incorporation rates of feedback from three sources across genres were calculated respectively.

**Table 2:** Coding scheme for feedback type.

**Feedback categories**			**Examples**
Surface-level feedback	Meaning-preserving	Word	*Preposition errors- “In the age of” should be changed into “at the age of.”*
		Sentence	“*It's not the amount of time spent on the devices that matters. It's what the children receive while browsing the web.” Better to combine these two sentences into one*.
	Grammar		*Please check if the conjunction is missing*.
	Mechanics		*Please check the spelling of “nutrition.”*
Meaning-level feedback	Meaning-related	Word	“*Nevertheless” does not fit here in logic. Please change it to another word*.
		Sentence	*This sentence is not completely consistent with the content of the given material. Please have a check and revise it*.
		Paragraph	*The second point is not sufficiently developed. Please add more effective details*.

To enhance the reliability of coding, an experienced colleague was invited to code 30% of the data. The inter-coder agreement rates for surface-level feedback, meaning-level feedback, and feedback incorporation were 96.8, 95.1, and 100%, respectively. After the discrepancies were discussed and resolved, the researcher coded the rest of the data.

To answer Research Question 2, the interviews were transcribed verbatim by the researcher and then checked by the same colleague. There were two steps in coding the transcripts. In Step 1, the transcripts were read through for several times and any segment of interest was attached a descriptive label (e.g., revision strategies, cognitive operations). In Step 2, the “template organizing style” (Dörnyei, 2007) was followed in that all these preliminary codes were compared and then categorized into four deductive codes (i.e., behavioral engagement, cognitive engagement, affective engagement and learner belief) informed by previous frameworks (Han and Hyland, 2015; Han, 2017). The same colleague was invited to code 30% of the data. The inter-coder agreement rates were 100, 91.2, 95.8, and 100%, respectively. The disagreements were resolved and the researcher coded the rest of the data.

## Results

### Quantity and Uptake Rates of Three Sources of Feedback Across Genres

As Table 3 presents, both feedback quantity and uptake differed across genres. Overall, there were 138 feedback points in argumentation, 125 in narration, and 121 in exposition, yet the highest uptake rate (84.3%) was found in exposition, followed by 80% in narration, and 73.2% in argumentation.

**Table 3:** Quantity and uptake rate of feedback across genres.

**Argumentation**			**Automatic feedback**			**Peer feedback**			**Teacher feedback**			**Total**		
			**Num**.	**Uptake Num**.	**Uptake rate**	**Num**.	**Uptake Num**.	**Uptake rate**	**Num**.	**Uptake Num**.	**Uptake rate**	**Num**.	**Uptake Num**.	**Uptake rate**
Surface-level	Meaning-preserving	Lexical	13	8	61.5%	7	2	28.6%	8	7	87.5	28	17	60.7%
		Sentence	0	0	0	5	1	20%	5	5	100%	10	6	60%
	Grammar		28	17	60.7%	8	4	50%	22	22	100%	58	43	74.1%
	Mechanics		14	14	100%	1	1	100%	1	1	100%	16	16	100%
	Total		55	39	**70.9%**	21	8	**38.1%**	36	35	**97.2%**	112	82	**73.2%**
Meaning-level	Meaning-related	Lexical	0	0	0	1	0	0	8	7	87.5%	9	7	77.8%
		Sentence	0	0	0	9	6	66.7%	3	3	100%	12	9	75%
		Paragraph	0	0	0	2	1	50%	3	2	66.7%	5	3	60%
	Total		0	0	**0**	12	7	**58.3%**	14	12	**85.7%**	26	19	**73.1%**
Total			55	39	**70.9%**	33	15	**45.5%**	50	47	**94%**	138	101	**73.2%**
**Exposition**			**Automatic feedback**			**Peer feedback**			**Teacher feedback**			**Total**		
			**Num**.	**Uptake Num**.	**Uptake rate**	**Num**.	**Uptake Num**.	**Uptake rate**	**Num**.	**Uptake Num**.	**Uptake rate**	**Num**.	**Uptake Num**.	**Uptake rate**
Surface-level	Meaning-preserving	Lexical	9	3	33.3%	5	4	80%	18	18	100%	32	25	78.1%
		Sentence	0	0	0	1	1	100%	2	2	100%	3	3	100%
	Grammar		21	13	61.9%	6	5	83.3%	9	8	88.9%	36	26	72.2%
	Mechanics		13	13	100%	1	1	100%	0	0	0	14	14	100%
	Total		43	29	**67.4%**	13	11	**84.6%**	29	28	**96.6%**	85	68	**80%**
Meaning-level	Meaning-related	Lexical	0	0	0	6	5	83.3%	12	12	100%	18	17	94.4%
		Sentence	0	0	0	3	3	100%	12	12	100%	15	15	100%
		Paragraph	0	0	0	3	2	66.7%	0	0	0	3	2	66.7%
	Total		0	0	**0**	12	10	**83.3%**	24	24	**100%**	36	34	**94.4%**
Total			43	29	**67.4%**	25	21	**84%**	53	52	**98.1%**	121	102	**84.3%**
**Narration**			**Automatic feedback**			**Peer feedback**			**Teacher feedback**			**Total**		
			**Num**.	**Uptake Num**.	**Uptake rate**	**Num**.	**Uptake Num**.	**Uptake rate**	**Num**.	**Uptake Num**.	**Uptake rate**	**Num**.	**Uptake Num**.	**Uptake rate**
Surface-level	Meaning-preserving	Lexical	9	8	88.9%	4	3	75%	9	8	88.9%	22	19	86.4%
		Sentence	0	0	0	2	2	100%	4	4	100%	6	6	100%
	Grammar		34	24	70.6%	10	8	80%	17	15	88.2%	61	47	77.1%
	mechanics		13	12	92.3%	0	0	0	3	3	100%	16	15	93.8%
	Total		56	44	**78.6%**	16	13	**81.3%**	33	30	**90.9%**	105	87	**82.9%**
Meaning-level	Meaning-related	Lexical	0	0	0	1	1	100%	6	5	83.3%	7	6	85.7%
		sentence	0	0	0	2	1	50%	3	3	100%	5	4	80%
		paragraph	0	0	0	3	2	66.7%	5	1	20%	8	3	37.5%
	Total		0	0	**0**	6	4	**66.7%**	14	9	**64.3%**	20	13	**65%**
Total			56	44	**78.6%**	22	17	**77.3%**	47	39	**83%**	125	100	**80%**

*The bold values are uptake rates of feedback across three feedback sources, two feedback types,and three genres*.

In relation to feedback source, it was revealed that (1) automated feedback was provided most in narration (56) with the highest uptake rate (78.6%) but least in exposition (43) with the lowest uptake rate (67.4%); (2) peer feedback was provided most in argumentation (33) but least in narration (22), with the highest and lowest uptake rate in exposition (84%) and argumentation (45.5%), respectively; (3) for teacher feedback, the greatest amount (53) and the highest uptake rate (98.1%) were found in exposition, while the opposite was true in narration (47, 83%).

In terms of feedback type, it was found that the uptake rate of surface-level feedback ranged from 82.9% (narration) to 80% (exposition) and 73.2% (argumentation), while exposition displayed the highest rate (94.4%) of meaning-level one, followed by argumentation (73.1%), and narration(65%). Besides, meaning-level feedback was incorporated more highly than surface-level one in exposition while the contrary was true of narration; their uptake rates were similar in argumentation.

When feedback source and type were considered together, both similarity and differences appeared. At surface level, automated feedback was more provided than teacher feedback and peer feedback, while teacher feedback was most highly incorporated across genres; automated feedback was incorporated to the smallest extent except in argumentation. At meaning level, teacher feedback was more provided than peer feedback and was also more highly incorporated except in narration.

### Learner Engagement With the Three Sources of Feedback Across Genres

#### Behavioral Engagement

How learners behaviorally engaged with feedback can be explored by their revision operations (i.e., incorporation or rejection of feedback) and strategies.

In terms of revision operations (see Appendix 2), Liu, a typical high incorporator, took up all the feedback across genres, except for automated feedback in exposition (75%) and narration (88.9%). Next was Yang who incorporated all the automated feedback in argumentation and peer feedback in narration and all the teacher feedback across genres. The rejection of grammar feedback and meaning-level feedback respectively contributed to the low incorporation of peer feedback in exposition (50%) and argumentation (0%). Like Liu and Yang, Ge incorporated all the teacher feedback across genres. However, she displayed the lowest incorporation of automated feedback (42.1%) and peer feedback (54.5%) in general. Particularly, she incorporated only automated feedback on mechanics in argumentation and narration, while rejecting most of the surface-level peer feedback in argumentation and all the meaning-level one in narration.

Yao, who was moderate in feedback incorporation, accepted automated feedback most highly in narration (90.9%) but least highly in argumentation (62.5%), while both peer feedback and teacher feedback was accepted fully in exposition.

In contrast, Wang was a typical low incorporator. Specifically, she rejected most of automated feedback and lexical-related peer feedback at both surface and meaning levels in argumentation, leading to only 37.5% of automated feedback and 22.2% of peer feedback incorporated; in narration she incorporated 33.3% of peer feedback and 40% of teacher feedback, rejecting most of it on grammar. Ren was also a low incorporator, who incorporated automated feedback (50%) and meaning-level teacher feedback (25%) least highly in narration and peer feedback least highly in argumentation (50%).

Learners also varied their revision strategies. Some mainly resorted to their linguistic knowledge (Yao) or intuitions (Ren, Ge), while others drew on their linguistic knowledge in response to surface-level feedback and consulted the online dictionary (Liu, Wang) or the teacher (Yang) when responding to meaning-level one.

#### Cognitive Engagement

Cognitive engagement with feedback involved learners' awareness of feedback at noticing and understanding levels, meta-cognitive operations to regulate their mental efforts, and cognitive operations to process feedback and make revisions.

In terms of the awareness of feedback, all the learners except Ge reported the difficulty in understanding some of automated feedback across genres. For Ge, no difficulty reported doesn't mean that she didn't encounter any difficulty in processing feedback. Rather, she only attended to the feedback on mechanics and plural or singular forms while ignoring all the rest. She explained that she would like to wait until teacher feedback was provided. For the rest, difficulty in understanding was mainly caused by the inaccuracy of automated feedback on grammar and collocation. For example, learners felt confused about some *Pigai* feedback which tagged as erroneous what was correct (e.g., subject-verb disagreement diagnosed in “*what I have mentioned is just the tip of the iceberg*”). In response to *Pigai*'s identification of some expressions (e.g., “*no striking growth”*) as “Chinese English,” some learners simply expressed their disagreements, whereas others reported their failure to figure out in what way these expressions were inappropriate since no suggestion was offered.

The difficulty in understanding peer feedback was also reported due to its lack of specific details. For instance, Yang mentioned her confusion about “*Connectives are missing in some sentences”* which was too general for her to locate the problem.

Difficulty in understanding led directly to the rejection of the feedback, which functioned as the main cause for the lower incorporation of automated feedback and peer feedback than teacher feedback in general. Once the feedback was understood, meta-cognitive and cognitive operations were deployed to process feedback and come up with revisions (or not), which varied among learners.

As high incorporators, Liu and Yang didn't just simply accept feedback without thinking. Instead, they employed specific and extensive cognitive and meta-cognitive operations. Take the following teacher feedback (in bold) in exposition for example:

*But we should not be as blind as a bat, without noticing(*^***turn a blind eye to***^*) some social problems, such as the aging society and the aged-care issue(*^***the former involves the latter***^*)*.

Liu recalled that, enlightened by the feedback, she developed creative revisions below, after reading the sentence and the context for several times:

*But we should also divert our attention to some social problems, such as the shrinking workforce and the increasingly severe aged-care issues in the aging society*.

Similarly, Yang exerted various efforts to process feedback across genres. For instance, she resorted to syntactic analysis to show that “*leaving*” in “*More sophisticated technologies are used to develop new drugs, leaving few diseases uncured*” introduced the adverbial of the result, instead of being used in the wrong grammatical form as suggested by the peer feedback in exposition. When it comes to meaning-level feedback, more extensive efforts were made. To illustrate, in response to feedback concerning the logic of the plot in narration, Yang recalled her own experience to search for some details, which can be added to remove anything unreasonable about the plot, as specified below:

*The doubt in the feedback concerns why I had to change the class before I could live in school dormitory. I recalled that in my middle school, those who lived on campus had to be placed in one class for better supervision, so I had to be relocated to this specific class. And I added this detail to the essay*.

In other cases, the two learners even thought beyond the feedback. Specifically, due to the teacher's detection of a spelling error in narration (i.e., “*winter vocation*^***vacation***^”) which was missed in automated- and peer-feedback cycles, Liu said she would pay more attention to the spelling of similar-in-form words in future writing. For Yang, some surface-level teacher feedback (e.g., “*there is a saying that says,*^***goes***^” “*make their weight proper*^***keep the figure***^”) in exposition triggered her reflections on vocabulary knowledge and writing quality, as recalled below:

*The teacher's corrections rendered my original expressions more effective. I know those words suggested by the teacher, but I just couldn't think of them when writing. I guess I am really poor in putting my vocabulary into active use. And I begin to realize that an error-free essay is not necessarily good in quality*.

Yao, the modest incorporator, also displayed deep engagement across genres in that he extensively employed meta-cognitive strategies (i.e., planning, evaluating) to regulate his processing of feedback and revision. For example, in response to a piece of teacher feedback on grammar in argumentation, he planned how to revise and then evaluated the outcome of his revision, as recalled below:

*This feedback says that the subject of “spares” in “Saving those labors doesn't mean that our brains get lazy but spares more time.” is ambiguous and suggests separating it into two sentences. I thought about it and revised it into “Saving…lazy. Actually it spares more time.” Comparing these two versions, I found the revised was really better because it expressed what I mean more effectively*.

Sometimes, Yao chose to give up revision after careful weighing, as exemplified by the teacher feedback on the theme in narration below:

*The feedback says that the theme is not so closely connected with the plot. I think it makes sense, but my insufficient command of language prevented me from delivering what I mean in a satisfactory way. If I followed the feedback, I would have had to revise the plot holistically, which was too demanding for me*.

Being writer-oriented, Wang (one of the low incorporators) applied her L2 writing beliefs to justifying her decisions of rejection. Specifically, in response to surface-level peer feedback in argumentation, she would rather keep her original expressions if nothing was wrong grammatically. When it comes to meaning-level feedback in narration, she chose to follow her original idea. For example, she explained her rejection of the teacher feedback on adding some details to a particular part of the plot, as below:

*This part is less important, so no detail is necessary. The climax, which is directly related with the theme, is what follows, where there are details provided*.

As to the tense inconsistency in the story mentioned in both peer and teacher feedback, she explained that she used the present tense instead of the past one in some sentences for the lively description.

In contrast, the other low-incorporator (Ren) tended to rely on her intuition and self-knowledge. Specifically, she had such a strong faith in her own intuition that she followed it completely to process the feedback without bothering to seek external verification. For example, she rejected two peer corrections in exposition (i.e., changing “*affect the virus*” into “*be infected with the virus*” and “*the technology upgrades*” into “*the technology is upgraded*”), just because she didn't feel anything wrong about the words used in that way. Similarly, without probing into the teacher's corrective intention of changing “*The replaced jobs are some simple ones without using brain”* into “*…with the use of the brain*,” she saw no difference between the two and thus no necessity for revision.

Moreover, even if she agreed with the feedback, she would refuse the revision based on her self-belief. To illustrate, in response to the peer feedback on the lack of connection between sentences in argumentation, she justified the rejection this way:

*This is how I am used to thinking—expressing my point without providing many supporting details. I had to add something, but I just couldn't think of what to add within a short period of time*.

Another example is her response to the teacher feedback on the lack of important details in narration, presented below:

*I already put into words what I remember about my own experience. There was nothing more I could think of nor could I make up any details. Besides, I am not the one who would like to share personal feelings or experiences without reservation. So I had to leave it as it was*.

Different from these five learners, Ge displayed very limited engagement with feedback. On the one hand, she accepted some of automated and peer feedback on simple grammatical issues, without any form of regulation. For example, one piece of peer feedback for exposition suggests changing “*increased*” in “*the increased level of medical care*” to “*increasing*.” In response, she simply corrected it, while leaving untreated another error of the same kind “*the increased level of economic development”* which was missed in peer feedback. This suggests that Ge only dealt with individual errors separately without associating them with similar ones.

On the other, she engaged with teacher feedback in a perfunctory manner, even if she completely incorporated it in revision. Specifically, instead of exerting herself to work out better revisions, she sought for the easy way out. Take for instance a piece of meaning-level feedback (in bold) below on a sentence in exposition:

*Because China is now in a peaceful era, people's lives are stable and average life expectancy has increased, the increasing level of medical care has also brought security to people's lives*.^***The causal relationship is confusing, since you are analyzing the causes for the increased life expectancy in this essay***^.

In response, she revised as below by adding a “*so*” clause, without checking whether the revision was grammatically acceptable, let alone checking whether it made the causal relationship more logic:

*Because...... …, the medical level is constantly developing*, ***so*** *people feel more security in their lives*.

#### Affective Engagement

In what way learners engaged affectively with feedback can be examined in terms of their emotional reactions and overall attitudes toward it.

Learners generally reported no specific emotions upon receiving automated feedback. However, once they began processing it, two different emotional responses arose. One is concerned with the error detected. For instance, Liu felt shame about making some simple grammatical and spelling errors for they were “*silly errors*” which should have been avoided. Yet, Ren felt pleasant to correct those simple errors which were “*easy to correct at first sight*.” The other is related with feedback quality. Learners (except Ge) expressed doubts about the accuracy of automated feedback, such as “*tagging error-free words as grammatically wrong”* (Wang) or “*tagging infrequent expressions as Chinese English*” (Liu). There were also complaints about its lack of clarity. For instance, Yang held that some feedback in exposition and narration was “*not clear enough to follow*,” which made her feel “*worried*” and “*at a loss*.” Overall, learners valued its timeliness and efficient detection of obvious errors, but also doubted its reliability and thereby its effectiveness, considering it played a “*quite limited*” role in improving essay quality.

When receiving peer feedback and teacher feedback, learners were all filled with appreciation and gratitude, but varied their emotional reactions when processing it. In response to peer feedback, some learners had similar emotions across genres. For example, Liu felt pleased with it because it was “*relevant, helpful, and reliable*,” and Ren also expressed her willingness to have it and felt “*no trouble*” in making revisions based on it. However, others' emotions differed across genres. While expressing satisfaction with peer feedback for exposition and narration by such appraisals as “*relevant*,” “*specific*,” and “*unambiguous*,” Wang and Yang had issues with it for argumentation. Particularly, Wang felt both confused and surprised because the feedback amounted to “*rewriting*” in which her original idea was “*misunderstood*,” while Yang displayed a little disappointment because it was “*too general to be helpful*.” The reverse was true for Yao and Ge who felt more content with peer feedback for argumentation. Specifically, Yao felt pleasantly surprised with it on the logic connection within arguments, but considered feedback for the other genres “*less thought-provoking and practical*.” Ge also felt enjoyment about it which helped her to reflect on her own idea and ways to convey it, yet feedback for the other genres was “*less detailed*.” Overall, learners thought highly of peer review and peer feedback, for it provided “a chance to learn from each other” and “a new perspective to view essay quality.” Yet, some (e.g., Ren) complained that peer feedback quality was limited by peers' language proficiency.

Compared with peer feedback, teacher feedback was more admired and trusted for its quality and effectiveness. To illustrate, Liu considered it “*professional, multi-dimensional, and constructive*,” which benefit her a lot in revision; Wang expressed her greatest expectation for it due to its identification of errors and problems missed by automated feedback and peer feedback, and regarded it as “*a valuable reference”* in revision. The similar reason was given by Ren for her approval of teacher feedback. Other learners reported some specific experiences. For example, Yang showed admiration for it on word choice which rendered the expression “*idiomatic and appropriate,”* and Yao felt similarly about it on not only word choice but also development of ideas which made the meaning conveyed in a “*more logic and reader-friendly*” way. Besides, Ge felt especially impressed by its “*striking-home*” clarity on syntactic errors. Also impressive to her was the teacher's suggestion on making clear the relationship between the ideas before revision. Overall, all the learners greatly valued teacher feedback and believed it played a key and indispensable role in improving draft quality across genres. Yet, some intermediate-lower learners expected it (especially on content) to be more specific in suggestion and more accessible in phrasing.

## Discussion

This study mainly explored learners' engagement with automated, peer and teacher feedback across three genres of L2 writing. It was found that the quantity and uptake rate of feedback in general, across three sources and two types all varied by genres, which adds finer-grained quantitative evidence to the effect of genres on learner engagement found qualitatively before (Han, 2019; Tian and Zhou, 2020). Particularly, peer feedback was provided least in narration but incorporated most highly in exposition, which was consistent with previous findings in L2 Spanish writing (Sánchez-Naranjo, 2019); surface-level feedback was incorporated less highly than meaning-level one in exposition, while incorporation rates of the two types of feedback were quite similar in argumentation, contrasting previous findings respectively (Dressler et al., 2019; Fan and Xu, 2020; Saeli and Cheng, 2021).

Across genres, at surface level, teacher feedback was most highly incorporated and only secondary to automated feedback in amount. This not only echoes previous findings that teacher feedback was incorporated more than peer feedback (Yang et al., 2006; Zhao, 2010; Dressler et al., 2019), but also adds support to the finding that with automated feedback provided, surface-level teacher feedback was still provided (Wilson and Czik, 2016; Jiang et al., 2020), which may be attributed to the low precision rate of automated feedback in identifying errors (Bai and Hu, 2017). This suggests the inadequacy of automated feedback and peer feedback in addressing linguistic problems with L2 writings across genres, contrasting findings of Tian and Zhou (2020), and thus lends further empirical evidence to the argument that teacher feedback can only be supplemented rather than replaced by either automated feedback or peer feedback in the L2 writing classroom (Yang et al., 2006; Bai and Hu, 2017; Jiang et al., 2020).

The investigation of how individual learners engaged behaviorally, cognitively, and affectively with feedback across genres uncovered the complexity of learner engagement, concurring with previous findings (e.g., Han and Hyland, 2015; Zhang and Hyland, 2018; Han, 2019; Tian and Zhou, 2020; Zhang, 2020). One of the contributors was the mediating effect of contextual and individual factors. Genre turned out to be a typical contextual factor, for learners differed in their behavioral engagement across genres as indexed by the degree of feedback incorporation. Another factor was feedback quality. For example, due to inaccuracy of some automated feedback and lack of clarity of some peer feedback, most learners encountered difficulty in understanding, which resulted in the lower incorporation of these two sources of feedback in revision. Individual factors, on the other hand, included language proficiency and learner belief. To illustrate, lower-proficiency learners either resorted to intuition and self-concept as an excuse to avoid revision (Ren) or perfunctorily dealt with feedback (Ge), which suggests the inhibiting role of lower language proficiency in learners' cognitive engagement, a phenomenon also found previously (Zhang and Hyland, 2018; Zheng and Yu, 2018); Wang justified her rejection of the feedback based on her L2 writing beliefs, indicating that rejection of feedback sometimes signals learners' creative and independent thinking in revision as well as confidence as a writer, as was also found before (Ferris, 1997; Yang et al., 2006; Mahfoodh, 2017).

Sometimes, contextual factors and individual factors come into effect together as evidenced in the finding that learners varied their emotional responses to feedback across sources and genres mainly by its perceived quality or usefulness, adding proof to the dynamic nature of emotions which were influenced by the interaction among individual learners, writing tasks, and feedback quality (Han and Hyland, 2019). For instance, learners varied their emotional responses to automated feedback by the nature of errors and feedback quality across genres. They doubted its accuracy and complained about its lack of clarity, which was also reported previously (Lai, 2010; Dikli and Bleyle, 2014; Zhang, 2016; Bai and Hu, 2017), resulting in a mixed attitude toward it. For peer feedback, despite some learners holding different emotions across genres, positive feelings were always associated with its relevance and clarity, while negative ones tended to be caused by misunderstanding or the lack of specification. Meanwhile, concerns were raised about its quality inhibited by language proficiency of peers. Similar distrust toward peers' language proficiency was identified (Saeli and Cheng, 2021), which was, however, not empirically supported in previous studies (Wu, 2019). Consistent with previous findings (Zheng and Yu, 2018), learner' emotions for teacher feedback were unanimously favorable, due to its identifying errors missed by automated or peer feedback, locating inappropriate word choice, as well as providing genre-specific suggestions on idea development and accurate diagnosis of syntactic errors. However, some of meaning-level feedback was considered by intermediate-lower learners as not detailed or accessible enough to follow, suggesting it might not be scaffolded adequately for these learners (Crosthwaite et al., 2020).

The other contributor was interconnectedness among three dimensions of learner engagement. For one thing, learners' understanding of feedback decided whether the feedback was incorporated or rejected while their cognitive and metacognitive processing conditioned how to incorporate it in revision; for another, learners varied their emotional responses to feedback across genres mainly by its perceived quality generated during cognitive processing. This suggests the influence of cognitive engagement on both behavioral engagement and affective engagement, which was also observed in automated feedback practice (Zhang, 2016). Moreover, similar to previous studies (Han and Hyland, 2015; Fan and Xu, 2020; Koltovskaia, 2020), the mismatch was uncovered between cognitive engagement and behavioral engagement in that low incorporation didn't necessarily go together with the lack of deep engagement (e.g., Wang) whereas high incorporation was not always associated with deep engagement (e.g., Ge).

## Conclusion

This study explored how six second-year English majors engaged behaviorally, cognitively and affectively with three feedback sources across three genres in a 16-week L2 English writing course. Results showed that across genres, differences existed in the quantity and incorporation rate of feedback in general, across feedback sources and feedback types; yet, surface-level teacher feedback remained the most highly incorporated. Moreover, individual differences were found in learner engagement with feedback. Behaviorally, learners differed in the incorporation rate of feedback across sources and genres and in revision strategies. Cognitively, some employed extensive cognitive and meta-cognitive processing of the feedback, whereas others didn't. Affectively, while some varied in their emotional responses to feedback across sources and genres, learners generally considered automated feedback as limited but thought highly of peer feedback and especially teacher feedback in improving draft quality. These differences reflected the complexity of learner engagement with feedback, which was triggered by (1) the mediating effect of contextual (genre, feedback quality) and individual factors (language proficiency, learner beliefs), and (2) the interconnectedness and inconsistencies among dimensions in that behavioral engagement was prompted by cognitive engagement which also exerted an influence on affective engagement on the one hand, and on the other, the mismatch existed between behavioral and cognitive engagements.

Adding to the research on learner engagement with feedback from a multi-dimensional perspective, this study built a detailed picture of how learners engaged with multiple feedback sources across genres. As such, it extended Tian and Zhou (2020)'s by shedding some light on the mediating effect of genres on learner engagement. Two pedagogical implications can be drawn from the findings to enhance learner engagement. Firstly, it is important to align contextual factors with individual learner ones (Han, 2019) in providing feedback. Particularly, for lower-proficiency learners, meaning-level feedback needs to be more specified and accessible, and the access to communicating with the peer and the teacher should be available to clarify the doubts or misunderstandings and to provide necessary scaffolding, especially in argumentation and narration; learners can also be trained to employ active strategies (e.g., using the corpus) to tackle problems with automated feedback on collocation. Secondly, it is necessary to integrate automated feedback with peer feedback and teacher feedback, during which the peers and the teacher could differ their focus by feedback type and genres. For example, peers should be more careful when giving feedback in argumentation; the teacher could pay more attention to the surface-level quality of expository and argumentative writings, and care should be taken when providing meaning-level feedback on narration.

Nevertheless, there are some limitations which should be acknowledged. First, given the homogeneous background of participants involved and the particular local context in which this study was situated, the generalizability of the findings should be viewed with caution. Participants from diverse backgrounds (e.g., linguistic, sociocultural) in various educational contexts can be included in future study. Second, with the focus on the target of feedback and its incorporation in revision, the study didn't systematically take into consideration the format and quality of feedback and revision, which can be introduced to future research to provide a finer-grained picture of learner engagement with feedback across genres. Another limitation lies in the use of a single method (i.e., stimulated-recall interview) for the elicitation of how learners thought and felt in response to the feedback. It would be ideal to adopt other methods (e.g., conversation analysis, action research) in future exploration. This study is also limited in that the feedback examined was all given online via a single technology (*Pigai*). Given the increasing use of technology-mediated feedback in L2 writing nowadays and the potential influence of technology on attributes of feedback (Loncar et al., 2021), attention should be paid to how learners engage with multiple feedback sources delivered via different or multiple affordances of technology (e.g., screencasting, word-processor, AWE), with a view to identifying the particular technology appropriate for the particular feedback source or instructional purpose. Last but not least, as emphasized by Han and Gao (2021) in their critical review of literature on learner engagement with written feedback, this line of research aims not only to “capture, explore and describe” learner engagement but also to ask “how and why” (p. 68). In this sense, the mechanisms underlying the complexity of learner engagement with multiple feedback sources across genres revealed in this study deserve further probing.

## Data Availability Statement

The original contributions presented in the study are included in the article/Supplementary Material, further inquiries can be directed to the corresponding author.

## Ethics Statement

Ethical review and approval was not required for the study on human participants in accordance with the local legislation and institutional requirements. The patients/participants provided their written informed consent to participate in this study.

## Author Contributions

The author confirms being the sole contributor of this work and has approved it for publication.

## Funding

The work was supported by the General Project of Philosophy and Social Science Research in Colleges and Universities in Jiangsu Province (2020SJA0878) and the Fundamental Research Funds for the Central Universities (JUSRP12061).

## Conflict of Interest

The author declares that the research was conducted in the absence of any commercial or financial relationships that could be construed as a potential conflict of interest.

## Publisher's Note

All claims expressed in this article are solely those of the authors and do not necessarily represent those of their affiliated organizations, or those of the publisher, the editors and the reviewers. Any product that may be evaluated in this article, or claim that may be made by its manufacturer, is not guaranteed or endorsed by the publisher.

## Footnotes

^1^Due to the outbreak of COVID-19 epidemic in early 2020, the course was given live on line via the Dingding software instead of off line in the classroom as it used to be.

^2^ http://www.pigai.org/

^3^As no meaning-preserving feedback was provided at the paragraph level, this sub-category was not included in coding.
