# Supplementary material for: Exploring Learner Engagement With Multiple Sources of Feedback on L2 Writing Across Genres
Source: Front Psychol. 2021 Oct 15;12:758867. doi: 10.3389/fpsyg.2021.758867 (PMC8554298; doi:10.3389/fpsyg.2021.758867)
Supplement: Supplementary file 1 [file Data_Sheet_1.docx]

**Appendix 1 Stimulated interview outline**

1. How were you feeling when you received the feedback?
2. I noticed you made a revision based on this feedback point. What were you thinking when you read it and used it for revision?
3. I noticed this feedback point but you didn’t take it. What were you thinking at that time?

**Appendix 2. Six learners’ feedback incorporation rates across genres**

|  |  |  |  | **Learner 1**  **(Liu)** | | | | **Learner 2**  **(Wang)** | | | | **Learner 3**  **(Yang)** | | | | **Learner 4**  **(Yao)** | | | | **Learner 5**  **(Ge)** | | | | **Learner 6**  **(Ren)** | | | |
| --- | --- | --- | --- | --- | --- | --- | --- | --- | --- | --- | --- | --- | --- | --- | --- | --- | --- | --- | --- | --- | --- | --- | --- | --- | --- | --- | --- |
|  |  |  |  | **A** | **E** | **N** | **G** | **A** | **E** | **N** | **G** | **A** | **E** | **N** | **G** | **A** | **E** | **N** | **G** | **A** | **E** | **N** | **G** | **A** | **E** | **N** | **G** |
| **Automatic**  **Feedback**  **%** | **SL** | **MP** | **Lex** | 100 | 100 | 100 | 100 | 50 | 33.3 | 100 | 62.5 | 100 | 0 | 100 | 75 | 50 | / | 100 | 80 | 0 | 0 | / | 0 | 0 | 0 | 0 | 0 |
|  |  |  | **Sent** | / | / | / | / | / | / | / | / | / | / | / | / | / | / | / | / | / | / | / | / | / | / | / | / |
|  |  |  | **Para** | / | / | / | / | / | / | / | / | / | / | / | / | / | / | / | / | / | / | / | / | / | / | / | / |
|  |  | **Grammar** | | 100 | 66.7 | 83.3 | 80 | 33.3 | 60 | 71.4 | 55.6 | 100 | 66.7 | 87.5 | 87.5 | 50 | 40 | 66.7 | 50 | 0 | / | 33.3 | 20 | 83.3 | 100 | 75 | 83.3 |
|  |  | **Mechanics** | | 100 | / | 100 | 100 | / | 100 | 75 | 87.5 | 100 | 100 | 100 | 100 | 100 | 100 | 100 | 100 | 100 | 100 | 100 | 100 | 100 | 100 | / | 100 |
|  |  | **Total** | | **100** | **75** | **88.9** | **88.5** | **37.5** | **66.7** | **78.6** | **64.7** | **100** | **50** | **90** | **85.7** | **62.5** | **70** | **90.9** | **75.9** | **33.3** | **66.7** | **42.9** | **42.1** | **77.8** | **75** | **60** | **72.2** |
|  | **ML** | **MR** | **Lex** | / | / | / | / | / | / | / |  | / | / | / | / | / | / | / | / | / | / | / | / | / | / | / | / |
|  |  |  | **Sent** | / | / | / | / | / | / | / |  | / | / | / | / | / | / | / | / | / | / | / | / | / | / | / | / |
|  |  |  | **Para** | / | / | / | / | / | / | / |  | / | / | / | / | / | / | / | / | / | / | / | / | / | / | / | / |
|  |  | **Total** | | / | / | / | / | / | / | / |  | / | / | / | / | / | / | / | / | / | / | / | / | / | / | / | / |
|  | **Total** | | | **100** | **75** | **88.9** | **88.5** | **37.5** | **66.7** | **78.6** | **64.7** | **100** | **50** | **90** | **85.7** | **62.5** | **70** | **90.9** | **75.9** | **33.3** | **66.7** | **42.9** | **42.1** | **77.8** | **75** | **60** | **72.2** |
| **Peer**  **Feedback**  **%** | **SL** | **MP** | **Lex** | / | / | / | / | 33.3 | 100 | / | 50 | / | / | 100 | 100 | / | 100 | / | 100 | 0 | / | / | 0 | / | 50 | 50 | 50 |
|  |  |  | **Sent** | / | / | 100 | 100 | 0 | 100 | 100 | 100 | / | / | / | / | 50 | / | / | / | 0 | / | / | 0 | 0 | / | / | 0 |
|  |  |  | **Para** | / | / | 0 | 0 | / | / | / | / | / | / | / | / | / | / | / | / | / | / | / | / | / | / | / | / |
|  |  | **G** | | / | / | 100 | 100 | 0 | 100 | 0 | 40 | / | 0 | 100 | 50 | 66.7 | / | 100 | 75 | 50 | 100 | 100 | 66.7 | / | 100 | 100 | 100 |
|  |  | **M** | | / | / | / | / | / | 100 | / | 100 | / | / | / | / | / | / | / | / | / | / | / | / | 100 | / | / | / |
|  |  | **Total** | | **/** | **/** | **75** | **75** | **25** | **100** | **33.3** | **52.9** | **/** | **0** | **100** | **75** | **60** | **100** | **100** | **71.4** | **33.3** | **100** | **100** | **50** | **50** | **75** | **80** | **72.7** |
|  | **ML** | **MR** | **Lex** | / | / | / | / | 0 | 100 | / | 50 | / | / | 100 | 100 | / | 100 | / | 100 | / | 100 | / | 100 | / | 66.7 | / | 66.7 |
|  |  |  | **Sent** | 100 | / | / | 100 | / | 100 | / | 100 | 0 | 100 | / | 50 | 100 | 100 | 100 | 100 | / | / | / | / | 33.3 | / | / | 33.3 |
|  |  |  | **Para** | / | / | 100 | 100 | / | / | / | / | / | / | 100 | 100 | 0 | 100 | 0 | 33.3 | / | 100 | 0 | 50 | 100 | 0 | / | 50 |
|  |  | **Total** | | **100** | **/** | **100** | **100** | **0** | **100** | **/** | **66.7** | **0** | **100** | **100** | **75** | **80** | **100** | **50** | **80** | **/** | **100** | **0** | **66.7** | **50** | **50** | **/** | **50** |
|  | **Total** | | | **100** | **/** | **80** | **83.3** | **22.2** | **100** | **33.3** | **55** | **0** | **50** | **100** | **75** | **70** | **100** | **66.7** | **76.5** | **33.3** | **100** | **50** | **54.5** | **50** | **62.5** | **80** | **63.2** |
| **Teacher**  **Feedback %** | **SL** | **MP** | **Lex** | 100 | 100 | / | 100 | / | 100 | 100 | 100 | 100 | 100 | 100 | 100 | 100 | 100 | 100 | 100 | / | 100 | / | 100 | 50 | 100 | 50 | 66.7 |
|  |  |  | **Sent** | / | 100 | / | 100 | / | / | 100 | 100 | / | / | 100 | 100 | 100 | 100 | / | 100 | / | / | 100 | 100 | 100 | / | / | 100 |
|  |  |  | **Para** | / | / | / | / | / | / | / | / | / | / | / | / | / | / | / | / | / | / | / | / | / | / | / | / |
|  |  | **G** | | 100 | 100 | / | 100 | 100 | 100 | 0 | 71.4 | 100 | / | 50 | 66.7 | 100 | / | 100 | 100 | 100 | 100 | 100 | 100 | 100 | 50 | 100 | 93.8 |
|  |  | **M** | | / | / | 100 | 100 | / | / | 0 | 0 | / | / | / | / | 100 | / | 100 | 100 | / | / | / | / | / | / | / | / |
|  |  | **Total** | | **100** | **100** | **100** | **100** | **100** | **100** | **50** | **81.8** | **100** | **100** | **83.3** | **94.1** | **100** | **100** | **100** | **100** | **100** | **100** | **100** | **100** | **92.3** | **75** | **85.7** | **87.5** |
|  | **ML** | **MR** | **Lex** | / | 100 | / | 100 | 50 | 100 | / | 100 | 100 | 100 | / | 100 | 100 | 100 | 100 | 100 | 100 | 100 | 100 | 100 | 100 | 100 | 50 | 83.3 |
|  |  |  | **Sent** | / | 100 | / | 100 | / | / | / | / | 100 | 100 | 100 | 100 | / | 100 | 100 | 100 | 100 | 100 | 100 | 100 | / | 100 | / | 100 |
|  |  |  | **Para** | / | / | 100 | 100 | / | / | 0 | 0 | 100 | / | / | 100 | 0 | / | 0 | 0 | / | / | / | / | 100 | / | 0 | 33.3 |
|  |  | **Total** | | **/** | **100** | **100** | **100** | **50** | **100** | **0** | **50** | **100** | **100** | **100** | **100** | **66.7** | **100** | **66.7** | **81.8** | **100** | **100** | **100** | **100** | **100** | **100** | **25** | **72.7** |
|  | **Total** | | | **100** | **100** | **100** | **100** | **75** | **100** | **40** | **73.3** | **100** | **100** | **85.7** | **96.3** | **92.3** | **100** | **93.3** | **94.6** | **100** | **100** | **100** | **100** | **93.8** | **87.5** | **63.6** | **82.9** |
